# Diseases of the Stomach

**Published:** 1899-08

**Authors:** 


Diseases of the Stomach. A text-book for practitioners and students. By Max Einhorn, M. D., Instructor in Clinical Medicine at the New York Post-Graduate Medical School and Hospital; Visiting Physician to the German Dispensary. Second Revised Edition. 502 pages. Price, cloth, $3.25 net; flexible leather, $3.75 net. New York: William Wood & Company. 1898.

It is only of late years that the diseases of the stomach have been thoroughly studied, yet more progress has been made in this field than in any other branch of internal medicine.

Dr. Max Einhorn's book is the first original work published in America which treats of this important subject, and as such it must fill a long-felt want. The new methods of diagnosis, which have made great advances of late, are here thoroughly and concisely described.

In discussing the different diseases of the stomach and their classification, the author shows great originality and has successfully endeavored to make the differential diagnosis as clear as possible.

The treatment and diet are fully given for each particular disease, and diet-lists stating the quantity of foods are often appended.

The book contains numerous original drawings of apparatus and microscopical specimens, thus greatly facilitating the conception of the text.

For every physician wishing to keep pace with the progress of the times the present book must be a welcome acquisition.

